# Beta Thalassemia Major with Gaucher’s Disease: A Rare Entity

**DOI:** 10.7759/cureus.5179

**Published:** 2019-07-20

**Authors:** Naila Bai, Sharmeen Nasir, Jawad Ahmed, Farheen Malik, Taha Bin Arif

**Affiliations:** 1 Pediatrics, Dow University of Health Sciences, Karachi, PAK; 2 Paediatrics, Dow University of Health Sciences, Karachi, PAK; 3 Internal Medicine, Dow University of Health Sciences, Karachi, PAK

**Keywords:** hemoglobinopathies, thalassemia, beta-thalassemia major, storage disorders, gaucher’s disease, protein-calorie malnutrition, anemic failure

## Abstract

Thalassemia is a genetic disorder due to deletion or mutation in the gene for alpha or beta chain of hemoglobin. Gaucher’s disease (GD) is characterized by a deficiency of a lysosomal enzyme, glucocerebrosidase which occurs due to mutations in the GBA1 gene on chromosome 1. Thalassemia and GD have overlapping clinical manifestations and present with features such as anemia, hepatosplenomegaly, and skeletal involvement. This creates a diagnostic conundrum for physicians. We present a case of an 11-month-old female who presented with fever, increasing paleness, and labored breathing. She had a recent history of uncross-matched transfusion. The child showed signs of anemic failure. Physical exam findings strongly pointed towards hemolytic anemia due to thalassemia major. Genetic analysis confirmed homozygosity in Fr 8-9 mutation confirming beta thalassemia major. Bicytopenia along with visceromegaly indicated malaria or storage diseases. Enzyme analysis revealed low levels of beta-glucocerebrosidase with normal acid sphingomyelinase levels confirming GD. In our case, we report the association of beta thalassemia major with GD which is a rare entity. The report highlights the need for an independent assessment of disorders that have similar presentations to avoid missing an associated disorder.

## Introduction

Thalassemia is one of the most prevalent genetic disorder in the world. It is due to deletion or mutation in the gene for alpha or beta chain of hemoglobin. Thalassemia intermedia and thalassemia minor are heterozygous conditions and are often referred to as beta thalassemia carrier or trait. Cooley’s anemia or Mediterranean anemia is synonymous with beta thalassemia major. Beta thalassemia is widespread in India, the Middle East, Central Asia, and Mediterranean countries. Southeast Asia and Cyprus have the highest beta thalassemia carrier frequency reported in the literature [1]. Thalassemia is the most widespread genetically influenced blood disorder in Pakistan. Milder or carrier form of thalassemia has a 5-7% prevalence in our country, with around 100,000 individuals affected with thalassemia major. This number is increasing, with 5-9,000 new cases are seen each year [2,3]. 

Gaucher’s disease (GD) has a global incidence of 1/57,000 to 1/75,000 live births [4]. It is rare, inherited, genetic deficiency of an important lysosomal enzyme, glucocerebrosidase. Mutations in the GBA1 gene, which is located on chromosome 1 (1q21) are responsible for the development of GD. Lack of this vital enzyme causes accumulation of undigested substrates, glucocerebroside, and glucosylsphingosine in the monocyte-macrophage system giving the characteristic Gaucher cells appearance [5]. Consequently, hepatosplenomegaly, thrombocytopenia, anemia, neurological impairment, and skeletal involvement appear as a clinical manifestation of the disease [6].

A thorough review of the literature revealed that different hemoglobinopathies coexist with storage disorders [7,8], but beta thalassemia major coexisting with GD is a rare entity. According to our literature search, no such case has been reported of this association till date. We find it imperative to report this case as the coexistence of these two disorders causes significant difficulty in diagnosing and treating such patients. We report a case from Pakistan of an 11-month-old female who presented to pediatrics emergency department (PED) with the complaints of high-grade fever, increasing paleness and labored breathing.

## Case presentation

An 11-month-old female vaccinated till date, resident of a rural area of Pakistan, was brought to PED in Karachi with complaints of fever, increasing pallor and difficulty in breathing for two weeks. There was no history of bleeding from any site but pallor gradually increased over time. Later, it was associated with severe difficulty in breathing. For these complaints, the child had a prior visit to the hospital once, where un-crossmatched pack cell volume (PCV) was transfused and then the child was taken home by the attendants. She was brought here when her condition deteriorated again. She was the fourth product of non-consanguineous marriage and the rest of the three elder siblings were alive and healthy. No significant family history of transfusions or expiry at early age or bleeding. She was developmentally appropriate for her age. Birth history was unremarkable.

On examination, she looked severely pale and cachexic with thin hair and coarse facies with frontal bossing. She was febrile, tachycardic, and tachypneic. Her weight and height were less than the third percentile for age. Anthropometric measurements showed low mid-upper arm circumference (8 cm) and fronto-occipital diameter (41 cm). She had abdominal distension due to massive and firm hepatosplenomegaly with a liver span of 10 cm and spleen was 8.5 cm in size. There was no lymphadenopathy. The central nervous system (CNS) and musculoskeletal examination were unremarkable.

A diagnosis of protein-calorie malnutrition (PCM) with congestive cardiac failure secondary to hemolytic anemia, specifically thalassemia major, was considered, because of the combination of anemia with visceromegaly, frontal bossing and the typical age of presentation. Leukemia and sickle cell were also considered as less likely differentials. The child was in critical condition and immediate management for anemic failure was started. Uncross matched PCV was transfused again with parental consent (cross-matching needed at least three hours). Transfusion was done after drawing samples for laboratory workup. Genetic mutation analysis for thalassemia and peripheral smear for sickle cell disease were sent.

Initial laboratory investigations showed severe microcytic, hypochromic anemia and thrombocytopenia. Hemoglobin (Hb) was 4.1 gm/dL and platelets were 96,000. After this report, two more PCV units (after cross-matching) were transfused, but despite transfusions, hemoglobin was not rising. Lactate dehydrogenase (LDH) was 746 U/L which was raised suggesting ongoing hemolysis. Peripheral smear was negative for sickle-shaped cells. Extended red cell antibody screening was performed which was positive for antibodies against antigen E and K. So, washed red blood cells (RBC) were then transfused under cover of steroid first and then under cover of intravenous immunoglobulins (IVIG). After this step hemoglobin improved.

As repeated complete blood counts (CBC) were showing persistent bicytopenia - anemia and thrombocytopenia (Table 1), along with visceromegaly, malaria and storage disorders - specifically GD or Niemann pick disease (NPD) were considered as coexisting differentials. Smear for malarial parasite done at three occasions turned out negative. Therefore, the enzyme analysis was sent for storage disorders.

**Table 1:** Complete blood count on different days showing progressive bicytopenia*, decreasing hemoglobin and platelets. RBC - red blood cell

Parameters	Investigation at Admission at Day 0	Investigation at Day 2	Investigation at Day 3	Investigation at Day 6
Hemoglobin* (mg/dL)	4.1	6.8	5.7	5.0
Hematocrit (%)	10.5	18.3	15.7	14
Mean cell volume (fL)	65.6	73.5	71.4	75
Total leukocyte count (x10^3^ /µL)	17.1	8.9	9.8	4.7
Platelets* (x10^3^ /µL)	96	96	82	55
RBC Morphology	Anistocytosis/ Hypochromia/ Microcytosis/ Macrocytosis	Hypochromia/ Microcytosis	Hypochromia/ Microcytosis	Normochromic/ Normocytic
Malarial Parasites	-	Not Seen	Not seen	Not seen

DNA mutation analysis for beta thalassemia major was found to be positive. The child was homozygous for Fr 8-9 mutation. On the other hand, the enzyme levels of beta-glucocerebrosidase done via Tandem mass spectrometry from dried blood spot were also low i.e., 0.7 µmol/L/hr (>2.5 - normal) which confirmed GD. Level of acid sphingomyelinase was found to be normal (result: 2.4 µmol/L/hr; > 0.9 - normal). Parents were reluctant towards bone marrow biopsy thus it was not performed. A final diagnosis of PCM with anemic failure secondary to beta thalassemia major coexisting with GD was made, along with alloimmunization.

The child was stabilized with transfusions and management for malnutrition was done. For the management of thalassemia, counseling was done regarding regular transfusions, iron chelation, and complications. Family screening for hemoglobinopathies was done on hematologist’s suggestion (Table 2). Unfortunately, for GD, due to non-affordability, enzyme replacement was not started until then. The patient was discharged at a Hb level of 10 gm/dL and platelets of 62,000. The patient is doing well in follow-ups.

**Table 2:** Family screening for hemoglobinopathies HbA - adult hemoglobin; HbF - fetal hemoglobin; M - male; F - female

Relation to Patient	Hemoglobin Types		
	HbA (%)	HbA2 (%)	HbF (%)
Mother	94.8	4.9	0.3
Sibling-1 M	97	2.7	0.21
Sibling-2 F	94.2	5.1	0.7
Sibling-3 M	94.6	5.1	0.4

## Discussion

The thalassemia syndromes are a heterogeneous group of disorders caused by inherited mutations that decrease the synthesis of either the alpha globin or beta globin chains that compose adult hemoglobin, leading to anemia, tissue hypoxia, and red cell hemolysis related to the imbalance in globin chain synthesis. Mutations that diminish the synthesis of beta globin chains lead to beta thalassemia.

Beta thalassemia major is usually presented with fatigue, feeding problems, weakness, a pale or yellow appearance of the skin, irritability, slow growth, distended abdomen, deformities in facial bones, and failure to thrive [9]. It is observed that thalassemia is more prevalent in consanguineous marriages [10], in contrast, our patient was not a product of consanguinity.

Children with beta thalassemia major have abnormal linear growth (stunting), varying degrees of muscle wasting, and altered anthropometric measures related to their age [11]. One study found that 60% of thalassemics are underweight [12]. Under-nutrition causes growth disturbances in children, especially in those suffering from thalassemia major. Malnutrition is primarily caused by inadequate nutrient intake and defective intestinal absorption leading to a decreased capacity to gain appropriate weight. Correspondingly on physical exam, the height, weight, and anthropometric measurements of our patient were lower than normal. Beta-thalassemia presents with distinct characteristics such as including frontal bossing and massive hepatosplenomegaly. Likewise, our patient also presented with similar features.

The clinical diagnosis of thalassemia major is made by red blood cell indices, peripheral blood smear, qualitative and quantitative hemoglobin analysis through electrophoresis, and more certainly through genetic analysis by PCR based procedures [9]. Quantitatively thalassemias can be defined as hemoglobin less than 7gm% (<7g/dl) with fetal hemoglobin between 70-90% and adult hemoglobin between 10-30% on electrophoresis at the time of diagnosis [9]. The laboratory workup of this patient demonstrated altered red cell indices and a microcytic hypochromic RBC in peripheral smear. Molecular and genetic analysis reveal that thalassemia major is caused by point mutations leading to abnormal RNA transcription or nonsense mutations producing an abnormal protein [13]. Genetic analysis of our patients confirmed beta-thalassemia major (Fr 8-9 mutation).

The child presented with fever, hepatosplenomegaly, and symptoms of anemia, the diagnostic reports did not show any sign of malaria despite being an endemic infectious disease in Pakistan [14]. It is observed that sickle cell anemia present concurrently or in a similar manner to thalassemia [15], however, diagnostic evaluation for sickle cell disease was negative in our case. Presence of antibodies against antigen E and K can be attributed to transfusion with unmatched PCV. Workup for inborn errors of metabolism was done and found to be positive for GD.

GD is an autosomal recessive genetic disorder in which a sphingolipid glucocerebroside accumulates in cells and various body organs due to deficiency of a lysosomal enzyme 'beta-glucocerebrosidase' [5]. The presentation of GD varies with its types but usually, it is manifested in children as hepatosplenomegaly, bone, cardiac or neurological involvement, and ocular signs [16]. Type 1 GD is the most common form of disease making up roughly 95% of patients in western countries. Symptoms include the spleen and liver enlargement, bone problems, and fatigue and it is usually distinguished by the absence of neurological impairment. Type 2 usually affects infants and manifest with neurological symptoms while type 3 (juvenile or subacute neurological GD) is a transitional form between type 1 and 2 GD [6]. Although a wide spectrum of phenotypes is seen in patients with GD, the hematologic manifestations may be similar to thalassemia resulting in diagnostic quandary [8].

Diagnosis of GD is suspected on the clinical history and physical examination. Confirmation is done by laboratory tests and genetic mutation studies showing deficient enzyme. The laboratory tests of this patient depicted deficient beta-glucocerebrosidase (0.7 µmol/L/h) and normal acid sphingomyelinase enzyme levels which ruled out Niemann pick disease. Diagnosis of GD is also made by seeing Gaucher’s cells on bone marrow aspirate. On light microscopy or periodic acid-Schiff (PAS) staining, Gaucher cells appear as oval or round cells with striated cytoplasm described as 'wrinkled tissue paper appearance' and eccentrically placed nucleus [16]. Unfortunately, bone marrow biopsy could not be performed in our case due to lack of parent’s consent. It can be concluded that the child was suffering from PCM secondary to beta thalassemia major with concurrent GD and minor atypical antigen-antibody reactions.

Thalassemia is generally treated by regular blood transfusions adjunct to chelation therapy. Latest therapies targeting different aspects of thalassemia are still under clinical trial [17]. While enzyme replacement therapy with alglucerase, velaglucerase alfa, or taliglucerase alfa are approved to ameliorate many symptoms of GD [18]. The child was managed symptomatically for PCM and thalassemia and was discharged with a better hemoglobin profile. The parents were also counseled about the child’s disease and further follow-ups were recommended.

## Conclusions

Thalassemia major and GD demonstrate a number of identical symptoms and laboratory features. Physicians should be vigilant and aware of the possibility of such rare associations for appropriate investigations, timely diagnosis, and management.

## References

[1] Flint J, Harding RM, Boyce AJ, Clegg JB (1998). The population genetics of the haemoglobinopathies. Baillieres Clin Haematol.

[2] Ansari SH, Shamsi TS, Ashraf M (2011). Molecular epidemiology of β-thalassemia in Pakistan: far reaching implications. Int J Mol Epidemiol Genet.

[3] Black M, Sinha S, Agarwal S, Colah R, Das R, Bellgard M, Bittles A (2010). A descriptive profile of β-thalassaemia mutations in India, Pakistan and Sri Lanka. J Community Genet.

[4] Meikle PJ, Hopwood JJ, Clague AE, Carey WF (1999). Prevalence of lysosomal storage disorders. JAMA.

[5] Beutler E, Grabowski GA (2001). From Gaucher disease. In the metabolic and molecular bases of inherited disease.

[6] Stirnemann J, Belmatoug N, Camou F (2017). A review of Gaucher disease pathophysiology, clinical presentation and treatments. Int J Mol Sci.

[7] Kini JR, Sreeram S, Hegde A, Kamath S, Pai RR (2017). Thalassaemia trait with Gaucher disease: a diagnostic dilemma. J Clin Diagn Res: JCDR.

[8] Miri-Moghaddam E, Velayati A, Naderi M, Tayebi N, Sidransky E (2011). Coinheritance of Gaucher disease and α-thalassemia resulting in confusion between two inherited hematologic diseases. Blood Cells Mol Dis.

[9] Galanello R, Origa R (2010). Beta-thalassemia. Orphanet J Rare Dis.

[10] Saeed U, Piracha ZZ (2016). Thalassemia: impact of consanguineous marriages on most prevalent monogenic disorders of humans. Asian Pac J Trop Dis.

[11] De Sanctis V, Soliman AT, Elsedfy H (2013). Growth and endocrine disorders in thalassemia: the international network on endocrine complications in thalassemia (I-CET) position statement and guidelines. Indian J Endocrinol Metab.

[12] Salih K, Al-Mosawy W (2013). Evaluation some consequences of thalassemia major in splenectomized and non-splenectomized Iraqi patients. Int J Pharm Pharm Sci.

[13] Sabath DE (2017). Molecular diagnosis of thalassemias and hemoglobinopathies: an ACLPS critical review. Am J Clin Pathol.

[14] Khattak AA, Venkatesan M, Nadeem MF (2013). Prevalence and distribution of human Plasmodium infection in Pakistan. Malar J.

[15] Lubega I, Ndugwa CM, Mworozi EA, Tumwine JK (2015). Alpha thalassemia among sickle cell anaemia patients in Kampala, Uganda. Afr Health Sci.

[16] Nagral A (2014). Gaucher disease. J Clin Exp Hepatol.

[17] Khandros E, Kwiatkowski JL (2019). Beta thalassemia: monitoring and new treatment approaches. Hematol Oncol Clin North Am.

[18] Zimran A, Elstein D (2014). Management of Gaucher disease: enzyme replacement therapy. Pediatr Endocrinol Rev.

